# The Impact of Tobacco Smoking on Adult Asthma Outcomes

**DOI:** 10.3390/ijerph18030992

**Published:** 2021-01-23

**Authors:** Angelica Tiotiu, Iulia Ioan, Nathalie Wirth, Rodrigo Romero-Fernandez, Francisco-Javier González-Barcala

**Affiliations:** 1 Department of Pulmonology, University Hospital of Nancy, 54511 Nancy, France; n.wirth@chru-nancy.fr; 2 Development, Adaptation and Disadvantage, Cardiorespiratory Regulations and Motor Control (EA 3450 DevAH), University of Lorraine, 54505 Nancy, France; ic.ioan@chru-nancy.fr; 3 Lung Function Testing Lab, Children’s University Hospital, 8 Rue du Morvan, 54511 Vandoeuvre-lès-Nancy, France; 4 Department of Medicine, Clinic University Hospital, Santiago de Compostela, 15706 Santiago de Compostela, Spain; rodrigo.romero.fernandez@sergas.es; 5 Department of Medicine, University of Santiago de Compostela, 15782 Santiago de Compostela, Spain; 6 Spanish Biomedical Research Networking Centre-CIBERES, 15706 Santiago de Compostela, Spain; 7 Department of Respiratory Medicine, University Hospital of Santiago de Compostela, 15706 Santiago de Compostela, Spain; 8 Health Research Institute of Santiago de Compostela (FIDIS), 15706 Santiago de Compostela, Spain

**Keywords:** tobacco smoking, asthma control, exacerbations, lung function, smoking cessation

## Abstract

**Background**: Tobacco smoking is associated with more severe asthma symptoms, an accelerated decline in lung function, and reduced responses to corticosteroids. Our objective was to compare asthma outcomes in terms of disease control, exacerbation rates, and lung function in a population of asthmatic patients according to their smoking status. **Methods**: We compared patients’ demographics, disease characteristics, and lung-function parameters in current-smokers (CS, *n* = 48), former-smokers (FS, *n* = 38), and never-smokers (NS, *n* = 90), and identified predictive factors for asthma control. **Results**: CS had a higher prevalence of family asthma/atopy, a lower rate of controlled asthma, impaired perception of dyspnea, an increased number of exacerbations, and poorer lung function compared to NS. The mean asthma control questionnaire’s (ACQ) score was higher in CS vs. NS and FS (1.9 vs. 1.2, *p* = 0.02). Compared to CS, FS had a lower rate of exacerbations, a better ACQ score (similar to NS), a higher prevalence of dyspnea, and greater lung-diffusion capacity. Non-smoking status, the absence of dyspnea and exacerbations, and a forced expiratory volume in one second ≥80% of predicted were associated with controlled asthma. **Conclusions**: CS with asthma exhibit worse clinical and functional respiratory outcomes compared to NS and FS, supporting the importance of smoking cessation in this population.

## 1. Introduction

Asthma is a disease characterized by chronic airway inflammation and defined by the presence of symptoms such as dyspnea, wheezing, cough, and chest tightness that vary over time and in intensity associated with variable expiratory airflow limitation. The prevalence of asthma is estimated at up to 18% in the general population. The goals of asthma management are to achieve good symptom control and decrease the risk of asthma-related mortality, exacerbations, and persistent airflow limitation, with minimal side-effects of treatment [1].

Approximatively 20% of asthmatics currently smoke, a prevalence comparable to that found in the general population [2,3,4]. Cigarette smoking in asthma patients is associated with more severe symptoms; increased healthcare use, costs (unscheduled doctor visits and frequent hospital admissions) and mortality; worse asthma-related quality of life; accelerated decline of lung function; and reduced response to inhaled corticosteroids, the cornerstone of controller treatments in asthma [2,5,6,7,8,9,10,11]. The need for smoking cessation is often emphasized in asthma-management guidelines, but recent data about the effects of active smoking and smoking cessation on asthma outcomes are lacking [5,12,13]. Nevertheless, some authors have not found any significant relationship between smoking and asthma control [3].

The principal aim of this study was to assess the influence of active tobacco smoking on asthma control, exacerbation rate, and lung-function parameters among three groups of asthmatic patients with different smoking histories (never-smokers, former-smokers, and current-smokers). The secondary aim was to identify the factors associated with asthma control in this population.

## 2. Materials and Methods

### 2.1. Study Design and Participants

This prospective observational study enrolled 176 adult asthmatic patients between September 2012 and May 2018 at the Department of Pulmonology of University Hospital of Nancy, France; and between January 2013 and July 2019 at the University Hospital of Santiago de Compostela, Spain. The diagnosis of asthma was established by a respiratory physician according to Global Initiative for Asthma (GINA) guidelines [1]. All patients had asthma symptoms and either an airflow reversibility (increase in forced expiratory volume in 1 s (FEV_1_) > 12% reported to baseline and 200 mL following inhalation of 400 µg salbutamol) or airway hyperresponsiveness (proved by a positive methacholine challenge test) [1,14]. All patients were receiving pharmacological treatment according to GINA guidelines [1]. Three groups were recruited during the scheduled visits: never-smokers (NS), former-smokers (FS), and current-smokers (CS). All participants gave written and signed informed consent, and the study was approved by the local ethics committee (identifier: R2017-21; Galicia 2013/457).

### 2.2. Protocol and Assessments

We collected demographic and clinical data including age, gender, body mass index (BMI), smoking status and assessment of tobacco exposure (estimated as number of pack-year), family history (personal atopy and asthma), presence of dyspnea quantified according to Modified Medical Research Council (mMRC) scale [15], asthma control [16], number of exacerbations per year, lung function parameters, the presence of psychological disturbances (anxiety or depression) as comorbidities of asthma [17], and controller medication use according to the five steps defined by GINA guidelines (therapeutic pressure) [1].

The pack-year was used to measure the tobacco exposure of a person over a long period of time; it was calculated by multiplying the number of packs of cigarettes smoked per day by the number of years the person has smoked. Atopy was documented by at least one positive skin prick test to an aero-allergen, in line with the European guidelines [18]. Dyspnea was evaluated according to the mMRC scale, a self-rating tool to measure the degree of disability that breathlessness poses on day-to-day activities: 0—no breathlessness except on strenuous exercise; 1—shortness of breath when hurrying on the level or walking up a slight hill; 2—walks slower than people of same age on the level because of breathlessness or has to stop to catch breath when walking at their own pace on the level; 3—stops for breath after walking ∼100 m or after few minutes on the level; and 4—too breathless to leave the house, or breathless when dressing [15]. Asthma control was assessed with the Asthma Control Questionnaire (ACQ) [16]. Controlled or partially controlled asthma was defined as an ACQ score ≤ 1.5, and uncontrolled asthma was defined as a score > 1.5 [19]. Asthma exacerbation was defined as a worsening of respiratory symptoms for more than 24 h and requiring treatment with systemic corticosteroids for at least three days, and was assessed for the previous year before inclusion in the study. The presence of anxiety and depression was assessed by the Hospital Anxiety Depression scale, and a score ≥ 8 for each psychological disturbance was considered as positive for the diagnosis [17].

Lung-function tests were performed using flow-volume curves and plethysmography to measure the FEV1, forced vital capacity (FVC), FEV1/FVC, total lung capacity (TLC), residual volume (RV), diffusion capacity for carbon monoxide (DLCO), and the ratio of DLCO and alveolar volume (KCO), all of which were expressed as a percentage of the predicted value (adjusted for age, gender, weight, stature, and ethnic background), and interpreted according to the current guidelines [20,21,22].

The most important parameters studied for the impact of tobacco-smoking exposure on asthma outcomes were asthma control (according to the ACQ score), the number of exacerbations per year, and lung-function parameters.

### 2.3. Statistical Analysis

Statistical analysis was performed using the SAS University Edition v9.4 software (SAS Institute, Cary, NC, USA). Quantitative variables were expressed as mean values ± standard deviation (SD), and qualitative variables as number (percentage). Comparisons among the three groups (NS vs. FS vs. CS) or between two groups (e.g., NS vs. FS, NS vs. CS, FS vs. CS) were performed using an ANOVA test and Tukey-Kramer procedure if the distribution was normal, or a non-parametric ANOVA, e.g., Kruskal–Wallis test, if the distribution was not normal. A non-parametric Wilcoxon–Mann–Whitney test was used for comparison between two groups in obese subjects or in normal-weight subjects. Qualitative variables were compared using a chi-square test or Fisher exact test as needed. Univariate logistic regression analyses were performed to assess the association of asthma control with patients’ characteristics. Independent variables that were associated with asthma control with a significance level of *p* < 0.2 in the univariate statistics were introduced into the multivariate model. Multivariate logistic regression analysis was performed using stepwise selection procedures, with an entry threshold of 0.2 and a stay threshold of 0.05. First-order interactions between the covariates were tested. The results are given as odds ratios (OR) with 95% confidence intervals. A *p* value < 0.05 was considered as statistically significant.

## 3. Results

### 3.1. Population Characteristics

An equal proportion of patients was enrolled in both clinics (51.1% of study population in France and 48.9% in Spain). The three study groups (NS, FS, and CS) were comparable in terms of age (more than 40 years old in mean), body mass index (BMI), the presence of personal atopy (more than 35% of patients in each group), age of asthma onset (more than half of patients in each group had late-onset asthma), and severity of dyspnea (between 1 and 2 on the mMRC scale), as shown in Table 1.

However, several differences were found. In the FS group, more than half of patients were males, and the prevalence of family atopy and anxiety as comorbidities of asthma were significantly higher compared to NS group. Compared to NS, the CS patients had higher prevalences of family asthma and atopy (40% vs. 20%, *p* = 0.008; respectively 33% vs. 18%, *p* = 0.030) and needed increased therapeutic pressure to control their asthma (treatment GINA step 3 ± 1 vs. 2 ± 1, *p* = 0.029) (Table 1). The mean of cigarette smoking was 14.4 ± 7.4 pack-years in the CS group and 5.3 ± 7.2 pack-years in the FS group, without a significant difference between these groups.

Due to the presence of the overweight/obesity factor in a high proportion of the study population, we performed a strata analysis according to BMI (Tables S1–S4). Asthmatic patients that were overweight/obese had similar characteristics to the patients with normal weight and the study population.

### 3.2. Asthma Outcome Measures

#### 3.2.1. Asthma Control

The number of patients with controlled asthma was significantly different between the groups, with 62 (73%) of NS patients, 21 (55%) of FS patients, and 20 (42%) of CS patients (Table 1). The mean ACQ score was comparable for NS and FS (1.2 ± 1.2 vs. 1.2 ± 1.1, *p* = 0.844) and inferior to 1.5, suggesting well/partially controlled asthma. In contrast, the CS had a mean score at 1.9 ± 1.1, suggesting uncontrolled asthma, and significantly higher compared to NS (*p* = 0.016) and to FS (*p* = 0.019) (Table 1). It is interesting to note that if the degree of the dyspnea was not significantly different between the groups (1.4 for NS and FS, and 1.9 for CS, *p* = 0.866), the number of patients describing this symptom was different. More than 70% of FS patients had dyspnea compared to only 38% of NS patients (*p* < 0.001) and 46% of CS patients (*p* = 0.020), without a significant difference between NS and CS (*p* = 0.360) (Table 1).

#### 3.2.2. Asthma Exacerbations

More than half of CS patients had at least one exacerbation per year requiring systemic corticosteroids, compared to 40% of FS patients (*p* = 0.004) and 22% of NS patients (*p* < 0.001), with no significant difference between NS and FS. The mean number of exacerbations per year was also significantly different between the groups, with the CS group having significantly more exacerbations per year compared to the NS or FS groups (*p* < 0.001), with no significant difference between NS and FS (Table 1).

#### 3.2.3. Lung Function

CS patients had lower lung-function parameters (FVC, FEV1) compared to NS and FS (Table 2). The mean value of FVC was significantly higher in NS than in FS (*p* = 0.024) and CS (*p* = 0.022). No significant difference was found between FS and CS (*p* = 0.935). Similarly, the mean value of FEV1 was significantly higher in NS patients compared to FS (93% vs. 83%, *p* = 0.011) and CS (93% vs. 79%, *p* = 0.001). The difference between FS and CS was not significant (*p* = 0.473).

The FEV1/FVC ratio was significantly lower for the CS and FS compared to NS (66% vs. 73%, *p* < 0.001; and 68% vs. 73%, *p* = 0.012) and inferior at 70%, suggesting a more important airflow limitation for asthmatic people exposed to tobacco smoking (Table 2). The mean values of TLC and RV were comparable for the three groups.

The DLCO mean value was significantly lower (but still normal) in the CS patients compared to NS (*p* = 0.002), but not significantly different compared to FS (*p* = 0.075). The KCO mean value was similar in the three groups (Table 2).

#### 3.2.4. Factors Influencing Asthma Control

According to the ACQ score, two groups were identified: one group including 107 patients with controlled asthma (ACQ score ≤ 1.5), and the second one including 69 patients having uncontrolled asthma (ACQ score > 1.5). Negative correlations (Pearson) were found between the number of pack-years and smoking status (r = −0.54, *p* = 0.020 for CS; r = −0.06, *p* non-significant for FS; and r = −0.35, *p* = 0.043 for CS plus FS).

The univariate analyses found several characteristics of patients having controlled asthma: age less than 65 years old, never-smoking status, no history of family asthma or atopy, no personal allergy, higher lung-function parameters (FEV1 ≥ 80% of predicted, FVC ≥ 80% of predicted, FEV1/FVC ≥ 70%, TLC ≥ 80% of predicted, DLCO ≥ 80% of predicted, KCO ≥ 80% of predicted), absence of dyspnea, anxiety or depression traits, and needs of treatments according to the first three steps of the GINA guidelines (Table 3).

Multivariate logistic regression analyses identified four factors associated with asthma control: never-smoking status, an absence of dyspnea and exacerbations, and the FEV1 ≥ 80% of predicted (Table 4).

Conversely, FS and CS had a lower chance to have controlled asthma (for the FS, by 10%, and for the CS, by 70%). Similarly, the patients having dyspnea or asthma exacerbations requiring systemic corticosteroids in the prior year were less likely to have controlled asthma (Table 4).

## 4. Discussion

This study provides both confirmatory and original data on the effects of active smoking on asthma patients compared to never-smokers and former-smokers. Compared to NS, CS had higher prevalence of family atopy and asthma, more frequently uncontrolled asthma with an increased mean of ACQ score, greater annual rate of exacerbations requiring systemic corticosteroids, poorer lung function, and lower diffusion lung capacity. The decision to quit cigarette smoking seems to attenuate this negative impact on asthma outcomes.

Previous studies showed that smoking has a negative impact on asthma control even though the criteria used to define the control was not always the same [6,23,24,25,26,27,28,29,30,31]. Several of them defined asthma control according to GINA criteria [6,24], others using national consensus guidelines [23], an asthma-control test [28], or ACQ scores [25,27,30]. By using the ACQ score to define the asthma-control level in the present study, we found that the proportion of asthmatic patients with controlled asthma was significantly lower in CS and FS compared to NS (42%, 55%, and 73%, respectively). In addition, the multivariate analysis showed that asthmatic CS patients were less likely to have controlled asthma by 70% compared to NS. The mean score in CS was significantly higher than that in NS and FS (1.9 vs. 1.2), indicating poorly controlled asthma in people who smoke (ACQ score > 1.5) [19], in line with other published data [25,30]. Taken together, these findings confirm a strong association between active smoking status and poor asthma control, as reported in population-based surveys from Switzerland [27], France [28], the UK [26,30], and the USA [29]. A previous study also found a significant dose-dependent relationship between pack-years and uncontrolled asthma. Compared to NS, asthmatic people who smoked more than 10 PA (like in our group CS) had an odds ratio of 13.38 (95% CI 4.57–39.19) to have uncontrolled asthma [32]. One possible explanation for the poor asthma control in patients who smoke could be that they are less sensitive to the therapeutic effects of inhaled corticosteroids, the most important controller treatment of asthma [8,9,33,34]. In concordance with previous data [25,30], the negative impact of smoking on asthma control seems to be at least partially reversible, as patients who had quit smoking reported significantly better asthma control than CS (a mean ACQ score in our study similar to NS), and had more chances to achieve the control by using an adequate medication (Table 1, Table 3, and Table 4). These findings must encourage physicians to offer smoking-cessation advice to all asthma patients who smoke, as also recommended by current guidelines [1]. The lack of association between asthma control and smoking mentioned by some authors may be affected by the “healthy smoker” effect [35].

It is interesting to note that in our study, a significantly lower proportion of patients described dyspnea in the CS group compared to FS (46% vs. 71%), suggesting the possibility of impaired perception of breathlessness by tobacco smoking in asthma patients, as previously reported [36,37]. Current data suggest that asthmatic patients have a low perception of dyspnea compared to healthy subjects, and this is more evident in patients with severe asthma [38,39,40,41]. In addition, a blunted perception of dyspnea has been identified as a risk factor for asthma exacerbations, hospitalizations, and fatal asthma attacks [41,42]. These findings could partially explain our results with higher rates of exacerbations in CS. However, the absence of dyspnea was identified as a predictive factor for asthma control in univariate and multivariate analyses (Table 3 and Table 4), probably because this symptom is recorded and quantified in the ACQ score.

Several studies assessed the effect of active smoking on exacerbations, and showed that adults with asthma who were CS had a higher risk of exacerbations than those who had never smoked [43,44]. Effectively, in our study the proportion of patients having exacerbations in the previous year was significantly increased in the CS group compared to NS and FS, and the mean number of asthma exacerbations per year in CS was twice as high in this group versus patients who never smoked or quit this habit (Table 1). According to our definition of exacerbations, the asthma patients who smoked had more frequently used rescue corticosteroid courses in the prior year compared to NS or FS. This has already been reported in severe asthma patients without significant difference between NS and FS [25]. These results suggest that smoking cessation could decrease the risk of asthma exacerbations and the need for systemic corticosteroids. Other published data showed that asthmatic patients who are active smokers have an increased number of life-threatening asthma attacks, higher mortality, more frequent episodes of work disability, unscheduled doctor visits, and hospital admissions, suggesting a greater severity of their exacerbations compared to non-smokers [45,46,47,48,49,50,51]. According to the results of our univariate and multivariate analyses (Table 3 and Table 4), patients without exacerbations in the previous year had more chances to achieve asthma control than those with exacerbations. This association was recently reported in a study that included asthmatic patients with a smoking history of at least 10 PA [6].

The mean values of FEV1 and FVC were significantly lower in the CS and FS groups compared to NS. It is known that people with asthma have a substantially greater decline in FEV1 over time than those without asthma [52], and smoking history is associated with an accelerated FEV1 decline independently of the presence of asthma [53]. Several studies showed that smoking and asthma had an additive effect on the decline in FEV1 with age [54,55,56]. The poorer lung function found in the CS group compared to NS and FS is probably the consequence of this additive effect, and is in line with other previous data [38,57]. In addition, one study showed that among patients with asthma, smoking history ≥10 PA is associated with an accelerated loss of lung function [56], and the mean level of the exposure in our study was 14 PA in the CS group. Even though FS have a worse lung function in the present study compared to NS, the mean values of FEV1 and FVC were greater than in the CS group and within normal limits (Table 2), supporting previous data that showed that asthmatic patients who quit smoking achieved a significant improvement of lung function [12] and a smaller decline in FEV1 if they continued to be treated with inhaled corticosteroids [58,59]. In addition, a FEV1 ≥ 80% has been identified as a predictive factor for well-controlled asthma (Table 4). The ratio of FEV1/FVC was significantly lower in CS compared to NS and FS, suggesting an increased airway limitation. This is due to the additive or synergic effect of the exposure to cigarette smoke on the inflammatory and remodeling processes in the asthmatic airways [2,35]. Similar results were found in other studies [5,38,59]. Indeed, previous data showed that CS with asthma have more airway and parenchymal abnormalities on high-resolution computer tomography (CT) scans [5,25,60]. The area of bronchial lumen measured on high-resolution CT scans was smaller in asthmatic smokers than in non-smokers [5], and there were more emphysematous changes in CS compared to NS or FS [23,25,60]. Even though in our study we did not have the imaging data, we found a lower lung diffusion capacity in asthmatic CS patients than in FS and NS, which is generally correlated to the presence of the emphysema on the CT scan [60,61]. Taken together, these findings in the CS group could suggest that several asthmatic patients developed features from chronic obstructive pulmonary disease, so they might have an overlap syndrome [1,62,63,64].

According to the univariate and multivariate analyses, several factors were associated with asthma control: the never-smoking status, the absence of dyspnea and exacerbations, and the FEV1 ≥ 80% of predicted. Most of these factors (except dyspnea) were identified by a recent study including asthma patients that were current and previous smokers with a history of ≥10 PA [6]. If the association between the active smoking status and the poor asthma control was well documented in different population-based surveys [24,26,27,28,29,30], the exacerbation-related events (oral corticosteroids use, unscheduled healthcare visits, and asthma hospitalizations) in the prior year as risk factors for uncontrolled asthma have been identified by only one study [29]. The association between the absence of dyspnea and asthma control has never been described before. Several of the factors associated with uncontrolled asthma found by our univariate analysis, such as age ≥65 years-old, personal allergy, and psychological disturbances, were already described in a large European cohort [24], while others such as family atopy and asthma, normal lung function parameters, and treatment needs by GINA 4 and 5 steps were identified for the first time. It is well known now that smokers perceive fewer benefits to treatment adherence than non-smokers, so later in their life they will need greater therapeutic pressure to control asthma symptoms [64]. Concerning psychological disturbances, smokers with asthma and higher levels of anxiety sensitivity are more likely to experience intense withdrawal symptoms and desire to smoke at the beginning of a quit attempt. They could benefit from smoking-cessation interventions that specifically target anxiety sensitivity, as well as prolonged use of nicotine replacement therapies to target withdrawal symptoms and cravings [65].

The strengths of this study are the inclusion of three groups of asthmatic patients with different tobacco-smoking histories and the study of the effects of cigarette smoking on the most important parameters of asthma outcomes: disease control, lung function, and exacerbation rate. However, there are some limitations in this study, in part because of its retrospective nature, a lack of an evaluation of the emphysema by high-resolution CT scan, and a lack of several biologic markers specific to asthma or smoking status.

## 5. Conclusions

Cigarette smoking is associated with worse asthma outcomes for a range of clinical and functional respiratory variables compared to never-smoking. The effect is more important for the asthmatic patients that are current-smokers than in those who decided to quit cigarette smoking. These findings must encourage physicians and asthmatic patients to adopt a no-smoking strategy in order to improve the long-term asthma outcomes in this population.

## Figures and Tables

**Table 1:** Clinical characteristics of subjects according to smoking status.

	NS	*p*	FS	*p*	CS	*p*	*p*-Value
	(*n* = 90)	NS vs. FS	(*n* = 38)	*FS vs. CS*	(*n* = 48)	NS vs. CS	NS vs. FS vs. CS
**Gender (male)**	28 (33%)	**0.010**	21 (55%)	0.500	23 (48%)	0.051	**0.020**
**Age (years)**	43 ± 13	0.247	46 ± 11	0.985	46 ± 17	0.267	0.370
**BMI (kg/m^2^)**	27 ± 6	0.104	29 ± 6	0.363	28 ± 7	0.565	0.330
**BMI *n* (%)**		0.210		0.320		0.910	0.480
Normal	44 (49%)		11 (29%)		21 (44%)		
Overweight	26 (29%)		14 (37%)		12 (25%)		
Obese	20 (22%)		13 (34%)		15 (31%)		
**Family history *n* (%)**							
Asthma	17 (19%)	0.210	11 (29%)	0.300	19 (40%)	**0.008**	**0.030**
Atopy	15 (17%)	**0.010**	14 (37%)	0.730	16 (33%)	**0.030**	**0.020**
**Personal atopy *n* (%)**	40 (44%)	0.400	20 (53%)	0.110	17 (35%)	0.310	0.270
**Number of pack-year**	0 ± 0	**<0.001**	5.3 ± 7.2	0.372	14.4 ± 7.4	**<0.001**	**0.004**
**Asthma onset *n* (%)**		0.283		0.170		0.283	0.571
Childhood	28 (31%)		9 (24%)		21 (44%)		
Adult	56 (62%)		29 (76%)		27 (56%)		
**Dyspnea**							
Presence *n* (%)	34 (38%)	**<0.001**	27 (71%)	**0.020**	22 (46%)	0.360	**0.003**
Degree mMRC	1.4 ± 1.3	0.820	1.4 ± 1	0.179	1.9 ± 1.1	0.335	0.866
**Asthma Control**							
Controlled *n* (%)	66 (73%)	**0.045**	21 (55%)	0.210	20 (42%)	**<0.001**	**0.001**
ACQ score	1.2 ± 1.2	0.844	1.2 ± 1.1	**0.019**	1.9 ± 1.1	**0.016**	**0.023**
**Exacerbations**		0.110		**0.004**		**<0.001**	**<0.001**
**No**	70 (78%)		23 (61%)		21 (44%)		
**1**	10 (11%)		9 (24%)		4 (8%)		
**≥2**	10 (11%)		6 (16%)		23 (48%)		
**Exacerbations number/year**	0.5 ± 0.1	0.195	0.6 ± 0.8	**0.002**	1.3 ± 0.2	**<0.001**	**<0.001**
**Comorbidities**							
Anxiety trait	9 (10%)	**0.040**	9 (24%)	0.930	11 (23%)	**0.040**	0.060
Depression trait	3 (3%)	**0.040**	5 (13%)	0.270	3 (6%)	0.420	0.110
**Treatment GINA steps**	2 ± 1	0.581	2 ± 1	0.162	3 ± 1	0.029	0.120

Note: NS: never-smokers; FS: former-smokers; CS: current-smokers; BMI: body mass index; mMRC: Modified Medical Research Council scale; ACQ: Asthma Control Questionnaire; GINA: Global Initiative for Asthma guidelines. Data is shown as mean ± standard deviation (SD). Bold characters show the significant differences (*p* value < 0.05).

**Table 2:** Lung-function parameters according to smoking status.

	NS (*n* = 90)	*p* NS vs. FS	FS (*n* = 38)	*p* FS vs. CS	CS (*n* = 48)	*p* NS vs. CS	*p*-Value Anova
FVC (L)	3.9 ± 1.1	0.896	4.0 ± 1.0	0.347	3.7 ± 1.3	0.308	0.540
FVC (% predicted)	109 ± 19	**0.024**	100 ± 22	0.935	100 ± 22	**0.022**	**0.010**
FEV1 (L)	2.9 ± 0.9	0.270	2.7 ± 0.8	0.418	2.5 ± 1.1	**0.042**	0.150
FEV1 (% predicted)	93 ± 21	**0.011**	83 ± 18	0.473	79 ± 25	**0.001**	**0.001**
FEV1/FVC (%)	73 ± 10	**0.012**	68 ± 10	0.449	66 ± 12	<**0.001**	**0.002**
TLC (L)	5.5 ± 1.8	0.751	5.6 ± 1.6	0.833	5.7 ± 1.6	0.911	0.890
TLC (% predicted)	104 ± 25	0.522	101 ± 19	0.361	105 ± 21	0.813	0.690
RV (% predicted)	118 ± 40	0.595	114 ± 28	0.683	117 ± 31	0.850	0.840
DLCO (%)	95 ± 16	0.153	91 ± 14	0.075	86 ± 10	**0.002**	**0.002**
KCO (%)	101 ± 15	0.987	100 ± 17	0.618	99 ± 12	0.533	0.790

NS: never-smokers; FS: former-smokers; CS: current-smokers; FVC: forced vital capacity; L: liter; FEV1: forced expiratory volume in one second; FEV1/FVC: ratio of forced expiratory volume in one second and forced vital capacity; TLC: total lung capacity; RV: residual volume; DLCO: diffusion lung capacity for carbon monoxide; KCO: ratio of DLCO and alveolar volume. Data is shown as mean ± standard deviation (SD). Bold characters show the significant differences (*p* value < 0.05).

**Table 3:** Variables related to asthma control identified by univariate analysis.

	Controlled (*n* = 107)	Uncontrolled (*n* = 69)	*p* Value
**Age, *n* (%)**			**0.002**
<35 years old	23 (57.5)	17 (42.5)	
35–44 years old	38 (77.6)	11 (22.4)	
45–54 years old	27 (56.3)	21 (43.8)	
55–64 years old	14 (70.0)	6 (30.0)	
≥65 years old	5 (26.3)	14 (73.7)	
**Smokers, *n* (%)**			**0.001**
NS	66 (73.3)	24 (26.7)	
FS	21 (55.3)	17 (44.7)	
CS	20 (41.7)	28 (58.3)	
**Gender, *n* (%)**			0.134
Male	39 (54.2)	33 (45.8)	
Female	68 (65.4)	36 (34.6)	
**BMI, *n* (%)**			0.345
Normal weight	46 (63.0)	27 (37.0)	
Overweight	33 (66.0)	17 (34.0)	
Obesity	28 (52.8)	25 (47.2)	
**Family asthma, *n* (%)**			**0.008**
Yes	21 (44.7)	26 (55.3)	
No	86 (66.7)	43 (33.3)	
**Family atopy, *n* (%)**			**0.003**
Yes	19 (42.2)	26 (57.8)	
No	88 (67.2)	43 (32.8)	
**Personal allergy, *n* (%)**			**0.034**
Yes	40 (51.9)	37 (48.1)	
No	67 (67.7)	32 (32.3)	
**Dyspnea, *n* (%)**			**<0.001**
Yes	25 (30.1)	58 (69.9)	
No	82 (88.2)	11 (11.8)	
**Exacerbations, *n* (%)**			**<0.001**
Yes	18 (29.0)	44 (71.0)	
No	89 (78.1)	25 (21.9)	
**Anxiety trait, *n* (%)**			**<0.001**
Yes	9 (31.0)	20 (69.0)	
No	98 (66.7)	49 (33.3)	
**Depression trait, *n* (%)**			**0.019**
Yes	3 (27.3)	8 (72.7)	
No	104 (63.0)	61 (37.0)	
**FEV1%, *n* (%)**			**<0.001**
<80%	17 (28.8)	42 (71.2)	
≥80%	90 (76.9)	27 (23.1)	
**FVC%, *n* (%)**			**<0.001**
<80%	1 (6.3)	15 (93.8)	
≥80%	106 (66.3)	54 (33.8)	
**FEV1/FVC, *n* (%)**			**<0.001**
<70%	38 (46.3)	44 (53.7)	
≥70%	69 (73.4)	25 (26.6)	
**TLC%, *n* (%)**			**0.020**
<80%	14 (87.5)	2 (12.5)	
≥80%	90 (57.7)	66 (42.3)	
**RV%, *n* (%)**			0.372
<80%	10 (71.4)	4 (28.6)	
≥80%	93 (59.2)	64 (40.8)	
**DLCO%, *n* (%)**			**0.040**
<80%	18 (46.2)	21 (53.8)	
≥80%	87 (64.4)	48 (35.6)	
**KCO%, *n* (%)**			**0.048**
<80%	9 (90)	1 (10)	
≥80%	96 (58.5)	68 (41.5)	
**Treatment GINA steps, *n* (%)**			**<0.001**
GINA 1	43 (87.8)	6 (12.2)	
GINA 2	22 (53.7)	19 (46.3)	
GINA 3	32 (64.0)	18 (36.0)	
GINA 4	3 (18.8)	13 (81.3)	
GINA 5	7 (35.0)	13 (65.0)	

NS: never-smokers; FS: former-smokers; CS: current-smokers; BMI: body mass index; FVC: forced vital capacity; L: liter; FEV1: forced expiratory volume in one second; FEV1/FVC: ratio of forced expiratory volume in one second and forced vital capacity; TLC: total lung capacity; RV: residual volume; DLCO: diffusion lung capacity for carbon monoxide; KCO: ratio of DLCO and alveolar volume; GINA: Global Initiative for Asthma guidelines. Bold characters show the significant differences (*p* value < 0.05).

**Table 4:** Factors associated with asthma control identified by multivariate analysis.

	OR * (95% CI)	*p* Value
**Smokers**		**0.030**
NS	1	
FS	0.9 (0.3–2.6)	
CS	0.3 (0.1–0.9)	
**Presence of dyspnea**	0.1 (0.03–0.3)	**<0.001**
**Exacerbations**	0.2 (0.1–0.5)	**<0.001**
**FEV1 ≥ 80% of predicted**	1.1 (1.02–1.07)	**<0.001**

* Adjusted by age, gender, BMI, family asthma and atopy, personal allergy, lung function, anxiety and depression traits; NS: never-smokers; FS: former-smokers; CS: current-smokers; FEV1: forced expiratory volume in one second. Bold characters show the significant differences (*p* value < 0.05).

## Data Availability

Not applicable.

## Supplementary Materials

The following are available online at https://www.mdpi.com/1660-4601/18/3/992/s1, Table S1: Clinical characteristics of subjects with BMI > 25 kg/m^2^ according to smoking status. Table S2: Lung function parameters of subjects with BMI > 25 kg/m^2^ according to smoking status. Table S3: Clinical characteristics of subjects with normal weight according to smoking status. Table S4: Lung function parameters of subjects with normal weight according to smoking status.
