# Supplementary material for: The Impact of Tobacco Smoking on Adult Asthma Outcomes
Source: Int J Environ Res Public Health. 2021 Jan 23;18(3):992. doi: 10.3390/ijerph18030992 (PMC7908240; doi:10.3390/ijerph18030992)
Supplement: Supplementary file 1 [file ijerph-18-00992-s001.pdf]

International Journal of Environmental Research and Public Health

MDPI

# Supplementary Material

**Table 1.** Clinical characteristics of subjects with BMI >25 kg/m² according to smoking status.

| | NS (*n* = 45) | *p* NS vs FS | FS (*n* = 27) | *p* FS vs CS | CS (*n* = 27) | *p* NS vs CS | *p* NS vs FS vs CS |
|---|---|---|---|---|---|---|---|
| **Gender (male)** | 19 (42%) | 0.430 | 14 (52%) | 0.590 | 12 (44%) | 0.850 | 0.720 |
| **Age (years)** | 42 ± 13 | **0.024** | 49 ± 11 | 0.180 | 54 ± 15 | **0.002** | **0.001** |
| **BMI (kg/m²)** | 31 ± 6 | 0.270 | 31 ± 5 | 0.930 | 32 ± 6 | 0.230 | 0.720 |
| **Family history *n* (%)** | | | | | | | |
| Asthma | 10 (22%) | 0.710 | 5 (19%) | 0.130 | 10 (37%) | 0.170 | 0.240 |
| Atopy | 8 (18%) | 0.240 | 8 (30%) | 0.340 | 5 (19%) | 1.0 | 0.450 |
| **Personal atopy *n* (%)** | 18 (40%) | 0.800 | 10 (37%) | 0.230 | 6 (22%) | 0.120 | 0.290 |
| **Number of pack-year** | 0 | **<0.001** | 10.5 ± 7.5 | 0.750 | 11.6 ± 9 | **<0.001** | **<0.001** |
| **Asthma onset *n* (%)** | | | | | | | |
| Childhood | 18 (40%) | 0.280 | 4 (15%) | 0.170 | 9 (33%) | 0.280 | 0.080 |
| Adult | 27 (60%) | | 23 (85%) | | 18 (67%) | | |
| **Dyspnea** | | | | | | | |
| Presence *n* (%) | 19 (42%) | **0.001** | 22 (81%) | **0.021** | 14 (52%) | 0.430 | **0.005** |
| Degree mMRC | 1.3 ± 1.1 | 0.632 | 1.8 ± 0.9 | 0.764 | 1.6 ± 1.2 | 0.555 | 0.780 |
| **Asthma Control** | | | | | | | |
| Controlled *n* (%) | 33 (73%) | 0.064 | 14 (52%) | 0.410 | 11 (41%) | **0.006** | **0.018** |
| ACQ score | 1.6 ± 1.3 | 0.235 | 1.2 ± 0.8 | **0.016** | 2 ± 1.2 | 0.380 | 0.053 |
| **Exacerbations** | | | | | | | |
| **No** | 36 (80%) | **0.012** | 14 (52%) | 1.0 | 14 (52%) | **0.012** | **0.014** |
| **1** | 5 (11%) | | 9 (33%) | | 4 (15%) | | |
| **≥2** | 4 (9%) | | 4 (15%) | | 9 (33%) | | |
| **Exacerbations number/year** | 0.3 ± 0.8 | **0.026** | 0.6 ± 0.7 | 0.43 | 1.0 ± 1.3 | **0.009** | **0.010** |
| **Comorbidities** | | | | | | | |
| Anxiety trait | 5 (11%) | 0.062 | 8 (30%) | 0.340 | 5 (19%) | 0.490 | 0.143 |
| Depression trait | 0 | **0.006** | 5 (19%) | 0.190 | 1 (4%) | 0.380 | **0.005** |
| **Treatment GINA steps** | 2 ± 1 | 0.620 | 3 ± 1 | 0.090 | 3 ± 1 | **0.029** | 0.067 |

NS : never-smokers ; FS : former-smokers ; CS : current-smokers ; BMI : body mass index ; mMRC : Modified Medical Research Council scale; ACQ : Asthma Control Questionnaire; GINA: Global Initiative for Asthma guidelines. Data was showed as mean ± standard deviation (SD). Bold characters for the *p* value <0.05

**Table 2.** Lung function parameters of subjects with BMI >25 kg/m² according to smoking status.

| | NS (*n* = 45) | *p* NS vs FS | FS (*n* = 27) | *p* FS vs CS | CS (*n* = 27) | *p* NS vs CS | *p* NS vs FS vs CS |
|---|---|---|---|---|---|---|---|
| FVC (L) | 4.0 ± 1.0 | 0.720 | 3.9 ± 1.1 | 0.093 | 3.4 ± 1.3 | **0.026** | 0.140 |
| FVC (% predicted) | 106 ± 19 | 0.170 | 99 ± 25 | 0.990 | 99 ± 24 | 0.270 | 0.300 |
| FEV1 (L) | 2.9 ± 0.9 | 0.130 | 2.6 ± 0.9 | 0.120 | 2.2 ± 1.1 | **0.007** | **0.011** |
| FEV1 (% predicted) | 92 ± 21 | 0.080 | 84 ± 20 | 0.420 | 77 ± 27 | **0.022** | **0.026** |

| | | | | | | | |
|---|---|---|---|---|---|---|---|
| FEV1/FVC (%) | 74 ± 10 | **0.025** | 68 ± 10 | 0.200 | 63 ± 13 | **0.001** | **<0.001** |
| TLC (L) | 5.8 ± 1.8 | 0.710 | 5.6 ± 1.6 | 0.660 | 5.5 ± 1.9 | 0.420 | 0.780 |
| TLC (% predicted) | 103 ± 22 | 0.410 | 98 ± 21 | 0.650 | 102 ± 23 | 0.930 | 0.710 |
| RV (% predicted) | 110 ± 45 | 0.510 | 112 ± 30 | 0.440 | 123 ± 37 | 0.150 | 0.410 |
| DLCO (%) | 96 ± 16 | **0.010** | 86 ± 11 | **0.046** | 92 ± 13 | 0.570 | **0.019** |
| KCO (%) | 103 ± 16 | 0.550 | 101 ± 16 | 0.720 | 100 ± 14 | 0.360 | 0.640 |

NS : never-smokers ; FS : former-smokers ; CS : current-smokers ; FVC : forced vital capacity ; L : litter ; FEV1 : forced expiratory volume in one seconde ; FEV1/FVC : ratio of forced expiratory volume in one seconde and forced vital capacity ; TLC : total lung capacity ; RV : residual volume ; DLCO : diffusion lung capacity for carbon monoxide ; KCO : ratio of DLCO and alveolar volume. Data was showed as mean ± standard deviation (SD). Bold characters for the *p* value <0.05

**Table 3.** Clinical characteristics of subjects with normal weight according to smoking status.

| | **NS (*n* = 45)** | ***p* NS vs FS** | **FS (*n* = 11)** | ***p* FS vs CS** | **CS (*n* = 21)** | ***p* NS vs CS** | ***p* NS vs FS vs CS** |
|---|---|---|---|---|---|---|---|
| **Gender (male)** | 9 (23%) | **0.024** | 7 (64%) | 0.710 | 11 (52%) | **0.022** | **0.014** |
| **Age (years)** | 45 ± 14 | 0.180 | 40 ± 10 | 0.570 | 37 ± 14 | **0.039** | 0.084 |
| **BMI (kg/m²)** | 22 ± 2 | 0.940 | 23 ± 1 | 0.350 | 22 ±26 | 0.430 | 0.560 |
| **Family history *n* (%)** | | | | | | | |
| Asthma | 6 (15%) | **0.014** | 6 (55%) | 0.530 | 9 (43%) | **0.019** | **0.012** |
| Atopy | 7 (18%) | **0.023** | 6 (55%) | 0.910 | 11 (52%) | **0.006** | **0.008** |
| **Personal atopy n (%)** | 20 (51%) | **0.033** | 10 (91%) | **0.049** | 11 (52%) | 0.940 | 0.053 |
| **Number of pack-year** | 0 | **<0.001** | 9.5 ± 6.9 | 0.340 | 11.9 ± 6.8 | **<0.001** | **<0.001** |
| **Asthma onset *n* (%)** | | | | | | | |
| Childhood | 17 (44%) | 0.740 | 4 (36%) | 0.260 | 12 (57%) | 0.320 | 0.460 |
| Adult | 22 (56%) | | 7 (64%) | | 9 (43%) | | |
| **Dyspnea** | | | | | | | |
| Presence *n* (%) | 13 (33%) | 0.490 | 5 (45%) | 0.720 | 8 (38%) | 0.710 | 0.750 |
| Degree mMRC | 0.8 ± 0.9 | 1 | 0.8 ± 0.9 | 1 | 0.8 ± 0.9 | 1 | 1 |
| **Asthma Control** | | | | | | | |
| Controlled n (%) | 29 (74%) | 0.480 | 7 (64%) | 0.260 | 9 (43%) | **0.016** | 0.054 |
| ACQ score | 0.7 ± 0.8 | 0.211 | 1.3 ± 1.5 | 0.392 | 1.8 ± 1 | **0.003** | **0.018** |
| **Exacerbations** | | | | | | | |
| **No** | 30 (77%) | 0.700 | 9 (82%) | **0.009** | 7 (33%) | **<0.001** | **<0.001** |
| **1** | 4 (10%) | | 0 | | 0 | | |
| **≥2** | 5 (13%) | | 2 (18%) | | 14 (67%) | | |
| **Exacerbations number/year** | 0.4 ± 0.8 | 0.820 | 0.4 ± 0.8 | **0.006** | 1.7 ± 1.3 | **<0.001** | **<0.001** |
| **Comorbidities** | | | | | | | |
| Anxiety trait | 4 (10%) | 1.0 | 1 (9%) | 0.370 | 6 (29%) | 0.140 | 0.180 |
| Depression trait | 3 (8%) | 1.0 | 0 | 0.530 | 2 (10%) | 1.0 | 0.840 |
| **Treatment GINA steps** | 2.2 ± 1 | 0.840 | 2.2 ± 1 | 0.590 | 2.4 ± 1 | 0.530 | 0.800 |

NS : never-smokers ; FS : former-smokers ; CS : current-smokers ; BMI : body mass index ; mMRC : Modified Medical Research Council scale; ACQ : Asthma Control Questionnaire; GINA: Global Initiative for Asthma guidelines. Data was showed as mean ± standard deviation (SD). Bold characters for the *p* value <0.05

**Table 4.** Lung function parameters of subjects with normal weight according to smoking status.

| | **NS (*n* = 45)** | ***p* NS vs FS** | **FS (*n* = 11)** | ***p* FS vs CS** | **CS (*n* = 21)** | ***p* NS vs CS** | ***p* NS vs FS vs CS** |
|---|---|---|---|---|---|---|---|
| FVC (L) | 3.9 ± 1.1 | 0.380 | 4.2 ± 0.8 | 0.810 | 4.1 ± 1.2 | 0.260 | 0.710 |
| FVC (% predicted) | 111 ± 19 | 0.052 | 102 ± 10 | 0.800 | 101 ± 20 | **0.046** | 0.073 |
| FEV1 (L) | 2.8 ± 1.0 | 0.900 | 2.8 ± 0.6 | 0.380 | 2.9 ± 1.1 | 0.390 | 0.950 |
| FEV1 (% predicted) | 95 ± 21 | **0.017** | 81 ± 13 | 0.440 | 83 ± 22 | **0.045** | **0.040** |
| FEV1/FVC (%) | 72 ± 10 | 0.170 | 67 ± 10 | 0.580 | 69 ± 11 | 0.400 | 0.430 |
| TLC (L) | 5.3 ± 1.9 | 0.380 | 5.7 ± 1.4 | 1.0 | 5.7 ± 1.1 | 0.110 | 0.540 |
| TLC (% predicted) | 104 ± 28 | 0.680 | 106 ± 9 | 0.710 | 108 ± 17 | 0.830 | 0.820 |
| RV (% predicted) | 127 ± 36 | 0.540 | 118 ± 22 | 0.130 | 108 ± 18 | **0.013** | 0.088 |
| DLCO (%) | 93 ± 15 | 0.110 | 86 ± 9 | 0.890 | 89 ± 15 | 0.150 | 0.240 |
| KCO (%) | 97 ± 14 | 0.840 | 99 ± 19 | 0.490 | 97 ± 10 | 0.540 | 0.910 |

NS : never-smokers ; FS : former-smokers ; CS : current-smokers ; FVC : forced vital capacity ; L : litter ; FEV1 : forced expiratory volume in one seconde ; FEV1/FVC : ratio of forced expiratory volume in one seconde and forced vital capacity ; TLC : total lung capacity ; RV : residual volume ; DLCO : diffusion lung capacity for carbon monoxide ; KCO : ratio of DLCO and alveolar volume. Data was showed as mean ± standard deviation (SD). Bold characters for the p-value <0.05
